# Advancing translational research for colorectal immuno-oncology

**DOI:** 10.1038/s41416-023-02392-x

**Published:** 2023-08-10

**Authors:** Elaine M. Thomas, Josephine A. Wright, Stephen J. Blake, Amanda J. Page, Daniel L. Worthley, Susan L. Woods

**Affiliations:** 1 Adelaide Medical School, The University of Adelaide, Adelaide, SA Australia; 2 Precision Cancer Medicine Theme, South Australian Health and Medical Research Institute, Adelaide, SA Australia; 3 School of Biomedicine, The University of Adelaide, Adelaide, SA Australia; 4 Lifelong Health Theme, South Australian Health and Medical Research Institute, Adelaide, SA Australia

**Keywords:** Cancer models, Colon cancer, Rectal cancer

## Abstract

Colorectal cancer (CRC) is a common and deadly disease. Unfortunately, immune checkpoint inhibitors (ICIs) fail to elicit effective anti-tumour responses in the vast majority of CRC patients. Patients that are most likely to respond are those with DNA mismatch repair deficient (dMMR) and microsatellite instability (MSI) disease. However, reliable predictors of ICI response are lacking, even within the dMMR/MSI subtype. This, together with identification of novel mechanisms to increase response rates and prevent resistance, are ongoing and vitally important unmet needs. To address the current challenges with translation of early research findings into effective therapeutic strategies, this review summarises the present state of preclinical testing used to inform the development of immuno-regulatory treatment strategies for CRC. The shortfalls and advantages of commonly utilised mouse models of CRC, including chemically induced, transplant and transgenic approaches are highlighted. Appropriate use of existing models, incorporation of patient-derived data and development of cutting-edge models that recapitulate important features of human disease will be key to accelerating clinically relevant research in this area.

## Introduction

Colorectal cancer (CRC) is a common and deadly disease. While resection and chemotherapy strategies have improved prognosis, it remains the second highest cause of cancer-associated death worldwide, accounting for 9.4% of cancer deaths [1]. Furthermore, the broad application of more recently developed approaches such as immunotherapy for this disease remains elusive, despite success in a variety of other solid tumours [2]. Immune checkpoint inhibitors (ICIs) such as anti-programmed cell death 1 receptor (anti-PD1) and anti-cytotoxic T-lymphocyte-associated protein 4 (anti-CTLA4) fail to elicit effective anti-tumour responses in the vast majority of CRC patients. This has been attributed to the T-cell depleted, DNA mismatch repair proficient (pMMR) microsatellite stable (MSS) CRC phenotype of the majority of cases [3]. Of all subtypes of CRC, the best responders to ICIs are DNA mismatch repair deficient (dMMR) and have microsatellite instability (MSI) [4]. This is thought to be due to the hypermutator phenotype and subsequent neoantigen production induced by defective DNA damage repair, allowing the tumour to be identified by the immune system ([5]). This subtype only accounts for 4–5% of patients with sporadic CRCs that progress to metastatic disease [6]. Initially, immunotherapy was exclusively offered as a late-line option for pre-treated metastatic MSI CRC patients [7]. However, several recent clinical trials have investigated immunotherapy as a frontline treatment for patients with MSI-high (H)/dMMR CRC [4], as opposed to chemotherapy. This is supported by the idea that exploitation of neoantigen-induced immunogenicity is most effective in earlier treatment lines or even earlier stages of CRC [8–10]. KEYNOTE-177 found significantly longer progression-free survival, fewer disease-related adverse events and improved quality of life in patients that received pembrolizumab (anti-PD1) compared to chemotherapy [11]. The Phase II CheckMate 142 Study combined pembrolizumab and ipilimumab (anti-CTLA4) to demonstrate durable clinical benefit using these immunotherapies as a frontline treatment for MSI-H/dMMR metastatic CRC patients, regardless of baseline demographic and tumour genetics [12]. While MSS/dMMR patients do not typically respond to ICIs, several novel strategies are showing promise. The inclusion of immunotherapy as a first-line treatment combined with FOLFOXIRI and bevacizumab improved progression-free survival in patients with previously untreated metastatic CRC [13]. Some efficacy has also been seen in pre-treated MSS/dMMR patients when immunotherapies were combined with temozolomide [14] and radiation [15]. Combining next-generation immunotherapy agents such as botensilimab and balstilimab have also shown promising clinical activity and durable response in heavily pre-treated metastatic CRC [16]. However, although these trials show promise and extend the period of disease control for some CRC patients, the majority of patients still eventually experience recurrence due to innate and acquired resistance.

**Fig. 1:**
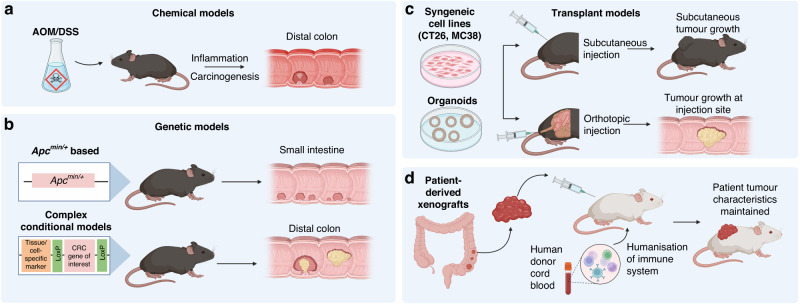
Colorectal cancer (CRC) mouse models used for immuno-oncology research. These currently used CRC models include **a** chemically induced, **b** genetic, **c** transplant models and **d** patient-derived xenografts.

Clearly, novel strategies are needed to improve immunotherapy outcomes in CRC patients. Before a new drug or therapeutic strategy enters the clinic, supporting evidence is usually first provided by preclinical testing. In vivo investigation, primarily in murine models, has led to dramatic improvements in the clinical landscape of many cancers. However, these success stories are few and far between; only a very small proportion of novel cancer drugs progress to approval, despite preclinical success [17]. A recent analysis of oncological clinical trial success rates in over 400,000 entries from 2000 to 2015, found only 3.4% of drug development programs advanced from Phase I to approval [17]. The lack of predictive value of current preclinical models is likely a key contributing factor to these low approval rates. Current murine CRC models have elucidated many of the underlying molecular mechanisms of CRC development and have proven responsive to ICI treatments [18, 19]. However, we remain unable to generate broadly acting and enduring ICI responses for CRC in the clinic [20]. This points to a need for a more comprehensive understanding of the CRC tumour immune microenvironment (TIME) and how the TIME influences ICI activity. If this is to be achieved, it is critical that we improve mouse models to mimic the TIME linked to the lack of reliable and enduring response to treatments, such as immunotherapy, as seen in the majority of CRC patients. This review summarises the current landscape of preclinical mouse models available (Fig. 1) and their advantages and shortfalls when applied to modelling immunotherapy responses.

## Chemically induced models of CRC

A primary chemical agent used for CRC induction in mice is azoxymethane (AOM). The metabolism of AOM into highly reactive alkylating species initiates tumorigenesis via mutation of genes in key signalling pathways such as the K-Ras, β-catenin and transforming growth factor-β pathways [21]. Many studies combine AOM with the colitis-inducing substance dextran sulphate sodium (DSS) to shorten tumour latency times and create an AOM/DSS colitis-associated model of CRC. This involves the formation of aberrant crypt foci, followed by adenoma and later carcinoma—the sequence of events also observed in human CRC [22]. This AOM/DSS model can be used to create syngeneic, immunocompetent models of spontaneous tumorigenesis that accumulate mutations over time in mice of various genetic backgrounds [23]. It is reproducible, potent, affordable and relatively straightforward to establish [24], and can be combined with genetically modified mouse models (GEMMs). The AOM/DSS model has been characterised as chromosomally unstable [25] and MSI [26], which is also observed in the early stages of chronic inflammation to colitis-associated cancer in humans [27]. However, while this model most accurately represents the inflammation–dysplasia–carcinoma pathway relevant to inflammatory bowel disease-associated CRC, this subtype only makes up 1–2% of CRC cases [28].

With regard to immunotherapy research, this model has been characterised as having a cytotoxic T-cell-infiltrated phenotype, as well as MSI [29], both properties typically associated with response to ICI in CRC patients. Surprisingly then, multiple studies report no significant therapeutic effect of anti-PDL1 or anti-CTLA4 immunotherapy in the AOM/DSS model [30, 31]. A convincing and broadly applicable rationale for this lack of response has not been forthcoming. This points to a potential use for this model in the context of modelling primary immunoresistance to ICI in MSI-H tumours, as observed in some patients [32, 33]. Otherwise, the AOM/DSS model has been used to investigate potential adverse immunotherapy-modulated effects on colitis-associated cancer patients, with Yassin and colleagues [34] finding that anti-PDL1 immunotherapy treatment induced significant weight loss in AOM/DSS-treated C57/BL6 mice, indicating systemic immune-mediated toxicity. Interestingly, this particular study attributed the lack of treatment effect on tumour growth to reduced infiltrating T cells in the late phase of tumour development. Alongside the challenges of this model, such as incomplete tumour penetrance, relatively long latency and variety in tumour antigens, the poor response to ICI treatment has limited its use as an immunotherapy screening tool [35]. However, this model has been shown to respond to alterations to the microbiome, rendering it a useful model for investigating the role of bacteria in the CRC TIME [36, 37]. Overacre et al. [36] recently discovered that colonisation of AOM/DSS tumour-bearing mice with an immunogenic bacterium, *Helicobacter hepaticus*, promoted anti-tumour immunity via induction of T follicular helper cells and peritumoural tertiary lymphoid structures. Therefore, while this model does have its limitations, it still represents a useful tool in CRC immunotherapy research, when used appropriately.

## Genetic models of CRC

GEMMs have been a cornerstone of cancer research for decades due to their utility in investigating the effects of one or multiple genetic alterations on cancer initiation and growth. These models can represent the spontaneous development of human tumours, in that they can develop de novo tumours gradually over time, in the presence of an intact immune system [38]. The most common CRC GEMMs are those based on alteration of *adenomatous polyposis coli* (*Apc*), due to the importance of *Apc* to CRC predisposition and sporadic CRC. The best-known *Apc* model is the Apc^*Min/+*^ mouse, which is considered a model of early MSS CRC. These mice develop many tumours, mostly located in the proximal intestine [39] and can be treated with chemical agents to accelerate neoplastic growth [40]. Additional mutations have been added over the years using Cre-lox recombination technology, for example, to localise tumorigenesis to the colon, with varying latency times and degree of tumour burden [41]. *Apc-*based models are commonly used to study familial adenomatous polyposis (FAP), as this disease is driven via hereditary *APC* mutations [42]. This allows for the observation of genotype-phenotype correlations and use as a preclinical model to test therapeutic options [43, 44]. In the context of CRC immune research, *Apc*-based GEMMs have been used to investigate immune populations in early CRC [45, 46], but are far less utilised for studies of immunotherapy treatment response, potentially due to the MSS nature of the model, with delayed and relatively high neoplastic burden. Furthermore, the fact that *Apc*^*Min/+*^ mice model early CRC limits their potential to test therapeutic strategies in advanced CRC, for which novel treatments are arguably needed the most.

Aside from *APC*, germline mutations in MMR genes such as *MLH1*, *MSH2*, *MSH6* and *PMS2* also predispose to CRC. Patients with these germline mutations are diagnosed with Lynch syndrome (LS), which accounts for ~3% of CRC patients. As the vast majority of LS tumours demonstrate MSI [47], LS patients were found to be some of the best responders to ICI therapy, which provided rationale to continued investigation [48, 49]. In the past, there has been difficulties developing LS mouse models, as germline inactivation of MMR genes such as *Msh2* predisposed mice to other diseases such as lymphoma [50, 51]. However, this limitation was overcome with the development of conditional *Msh2* knockout animals using a *Villin-Cre* transgene system (VCMsh2LoxP), which caused development of MSI tumours exclusively in the intestinal tract [52]. Since then, more complex and clinically relevant genetic models of LS have been developed [53]. These models of LS have been employed to investigate the feasibility of cancer vaccines based on recurrent frameshift neoantigens [54] as well as the effect of anti-cancer drugs such as aspirin [55], cisplatin and FOLFOX [52]. Although patients with LS-derived tumours generally respond well to ICIs, investigation into increasing efficacy and decreasing off-target toxicity remains important. Thus, there is value in the continued investigation of the immune landscape of these genetic models and response to novel immunotherapy strategies.

In recent years, advancements in gene editing technology and the concurrent discovery of additional important driver genes in CRC have allowed the development of increasingly complex CRC GEMMs that more accurately recapitulate late-stage CRC. For example, Tauriello, Palomo-Ponce [56] created a C57BL/6 mouse strain bearing conditional alleles of four key driver mutations in human CRC *(Apc*^*fl/fl*^*, Kras*^*LSL-G12D*^*, Tgfbr2*^*fl/fl*^ and *Trp53*^*fl/fl*^*)* in intestinal stem cells using a *Lgr5*^*eGFP-creERT2*^ driver. In total, 90% of quadruple-mutant mice developed tumours which had a highly stromal phenotype, T-cell exclusion and limited response to PDL1 inhibition, thus reproducing key features seen in advanced MSS CRC in humans. However, a key limitation of many GEMMs is that all cells (or, in the case of tissue-specific promoter systems, all cells of a specific cell type) contain the mutation(s) of interest, meaning potential concurrent carcinogenesis and reduced load of passenger mutations in cells [38]. Developments in gene editing approaches in situ have allowed somatic mutations to be introduced within the microenvironment of a colon that is genetically wild-type [57]. However, despite their use in modelling the pathogenesis of CRC, comparatively few studies use GEMMs as preclinical models to investigate immunotherapy efficacy. This may be due to several reasons. GEMMs are time-consuming, laborious, and expensive models to create, and maintenance of strains with multiple mutations can require complicated breeding strategies. They can also have prolonged latency periods and tumour establishment can be variable and difficult to monitor. Furthermore, although tumours contain key CRC driver mutations, they are not acquired sequentially or heterogeneously, resulting in cold, genetically stable cancers with low mutational burden that do not respond well to immunotherapy [58]. This is in a sense, a useful characteristic as the majority of CRC is nonresponsive to immunotherapy, and thus success in GEMM models may have more predictive value than other models, setting a high bar for novel therapies being tested.

## Implantation models of CRC

Implantation of either murine or human cells into mice is the most common method of establishing colorectal tumours [59]. These models are often applied to testing novel drugs and treatment strategies, owing to their feasibility and reproducible nature. CRC cell lines, organoids and tumour tissue (murine or human) can be implanted either subcutaneously or orthotopically, into the mucosa of the colorectum. To understand response in the presence of a functional immune system, immunocompetent syngeneic hosts are required. However, useful information concerning the evolution of tumour immune immunoediting may also be uncovered by comparison of tumour cells transplanted into immunodeficient and immunocompetent hosts.

### Conventional cell line models

In 2016, 83% of preclinical in vivo CRC studies published that year were derived from cell lines [59], and they remain popular tools. In CRC immunotherapy research, the two most used immune-competent syngeneic murine models are CT26 [60] and MC38 [61], which are of BALB/C and C57BL/6 murine strain origin, respectively. Both these cell lines were originally derived from chemically induced carcinomas and create robust tumours when injected at both ectopic and orthotopic sites [62]. These cell models are time efficient, cost-effective, and can be genetically modified to investigate properties of CRC immunogenicity such as MSI status and neoantigen presentation [8, 63]. MC38 has been shown to recapitulate MSI CRC, owing to a mutation in MMR gene *Msh3* [62, 64]. Notably, MC38 is highly immunogenic, has an immune-infiltrated phenotype, and is responsive to PD1/PDL1 blockade [65]. CT26 is highly undifferentiated and proliferative, and is often used as a MSS/pMMR experimental model due to its lack of mutations in MMR genes *Mlh1* and *Mlh2* [66]. However, neither MC38 nor CT26 have mutated *Apc*, which is mutated in the majority of human CRC. CT26 in particular has very little genetic similarity with human MSS CRC, sharing only mutated *Kras* amongst the most frequent drivers of human CRC [66]. Further, in a study comparing 10 different murine models, CT26 had tenfold higher cytolytic activity (defined in this study as the log average expression of two key cytolytic effectors, granzyme A and perforin) when compared to TGCA CRC data, and was the best responder to anti-CTLA-4 therapy [19]. This may be due to its relatively high tumour mutational burden and neoantigen load [19] owing to its alkylating agent-induced (N-Nitroso-N-methylurea) origins, which caused enrichment in C > T mutations [66]. However, CT26 tumours are less responsive to anti-PDL1/anti-PD1 therapy, with some variability in the literature. Dosset, Vargas [67] observed no response when an anti-PD1 antibody was used as a monotherapy to treat CT26 tumour-bearing Balb/c mice. In a separate study, this model was shown to be a partial responder to anti-PDL1 monotherapy [68], indicating that CT26 tumours may have an intermediate MSS and MSI phenotype. This variability may also be attributed to differences in the injected cell number, timing of treatment initiation and dosage of the treatment itself. This highlights a lack of consistency in immunotherapy treatment experiments in general, which hinders interpretation of results within the field as a whole.

Tumours derived from conventional cell lines lack the genomic and environmental heterogeneity typically observed in human tumours [35]. This is particularly important in CRC, owing to its heterogenous nature. In addition, carcinogen exposure and extended in vitro culture time are key players in the aetiology and pathogenicity of these lines. This is in sharp contrast to the sequential, adaptive, and heterogeneous accumulation of mutations in human CRC [6]. Thus, these cell lines should be used with caution when applied to CRC immunology research, particularly in regard to developing translational and clinically relevant immunotherapy strategies and targeted agents. For example, a recent Phase III clinical trial (IMblaze370) combining PD1 inhibition with MEK inhibition in previously treated, metastatic MSS CRC did not meet its primary endpoint of improving overall survival [69]. This clinical trial was supported by preclinical data in subcutaneous CT26 models that saw an increase in T-cell infiltration and augmented efficacy of PD1 inhibitors through increasing MHC-1 and PDL1 expression [68, 70]. This is just one instance of many that highlight the potential lack of clinical relevance of these models for immunotherapy research. However, while the field moves towards more accurate, but significantly more elaborate models, they serve as a starting point for proof-of-concept experiments of novel technologies and treatment options.

### Importance of tumour site

**Fig. 2:**
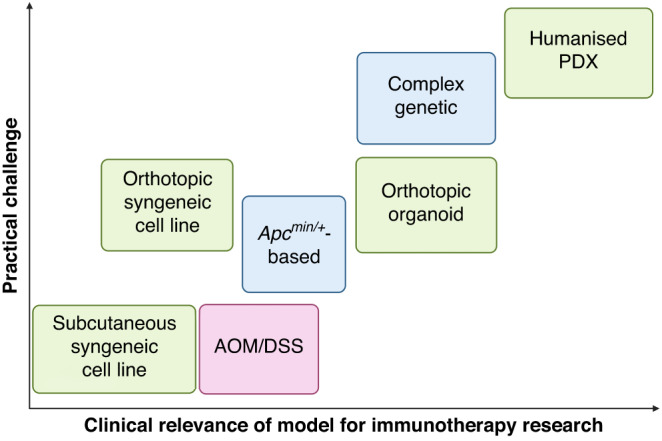
The clinical relevance of colorectal cancer models in immunotherapy research compared to the practicality of their establishment. Transplant (green), genetic (blue) and chemical (red) models are included.

Subcutaneous injection of tumour cells into ectopic sites such as the mouse flank is a common preclinical model of tumour establishment used across the field of oncology research. The popularity of this technique is not without reason; the establishment of a subcutaneous mouse tumour is relatively straightforward, and tumours can be easily monitored and accessed, facilitating tumour-specific administration of treatments. However, subcutaneous tumours do not create the same TIME as orthotopic tumours, as the stromal environment of the colonic mucosa cannot be accurately replicated in the epidermis through injection of tumour cells alone. Furthermore, accumulating evidence suggests that the TIME in which tumours are established has a marked effect on their growth and therapy response [71–73]. This calls into question the suitability of subcutaneous models for testing the efficacy of preclinical immunotherapies. For example, a study found that CT26 tumours had significantly different immune infiltrates when injected subcutaneously rather than into the caecum [74]. Furthermore, a recent study [62] demonstrated that CT26 and MC38 orthotopically injected into the colon and into the liver were significantly less responsive to immune checkpoint blockade compared to subcutaneous models using the same cell lines. The orthotopic tumours had significantly fewer T cells and dendritic cells, as observed in human pMMR/MSS CRC. Similar studies in other immunotherapy-resistant cancers, such as prostate cancer bone metastasis, have also illustrated that the TIME of tumours at the orthotopic site has characteristics that confer resistance to ICIs, as opposed to that of the subcutaneous TME [71]. As ICI therapy requires localisation of immune cells at the site of the tumour to be effective, evidence of success in subcutaneous models may be misleading. However, these studies investigate the mechanisms behind these site-specific differences in response, which may be useful in advancing our knowledge of the determinants of ICI responses. Furthermore, concurrent induction of orthotopic tumours with subcutaneous tumours has provided insight into the mechanisms of systemic immunosuppression mediated by CRC liver metastasis, with two studies finding that doing so reduced systemic anti-tumour immunity in mice compared to those with flank tumours alone [75, 76]. Hence, while subcutaneous tumours remain an important tool in immunotherapy research, orthotopic tumours likely provide a more accurate representation of the TME of CRC (Fig. 2).

### Use of organoid technology to generate CRC mouse models

The development of organoid technology has revolutionised oncology research, leading to more accurate physiological in vitro modelling of a variety of primary tissues compared to traditional 2D cell lines [77]. In recent years, genetic engineering advancements such as the development of CRISPR/Cas9 technology have allowed the rapid generation of CRC organoid lines that harbour specific mutations which mirror those commonly found in different subtypes of CRC. Injection of these organoids into mice creates genetically accurate CRC models in a manner that does not require exhaustive breeding strategies of multiple transgenic mouse lines. Organoid lines can also be isolated from GEMM tumours, which share the same genetic characteristics as the mouse line of origin, or from patient-derived tissue [78]. Implantation of organoids combines the efficiency of cell line models with the genetic accuracy of GEMMs (Fig. 2). They also allow versatility in their ability to be implanted at different physiological sites, such that tumour growth at both primary and metastatic sites can be investigated [56, 79]. A key caveat of implantation models, however, is that tumour cells do not arise de novo, thus the positioning of implanted cells can be aberrant with this technique. The versatility of CRISPR has meant that both human and murine organoid models of different CRC subtypes can be created, including serrated [80], MSS [56, 57] and MSI CRC [81]. This has facilitated investigation of specific molecular targets that modulate the TIME [79]. The use of these types of models is still in its infancy, but emerging studies have provided insight into determinants of CRC immunogenicity, such as tumour mutation burden. Westcott et al. [82] investigated tumour mutation burden in CRC through generation of sophisticated CRC orthotopic organoid murine models that expressed different levels of model neoantigen OVA. This study showed that low-level neoantigen expression in primary CRC may prevent T-cell priming and induce T-cell dysfunction. However, comparatively few studies use syngeneic mouse organoid engraftment to model therapy response, with the majority of studies opting to use cell lines, as previously discussed. Studies tend to combine in vitro patient-derived organoid co-culture screening to initially identify targets, and then move to cell line-derived syngeneic models to test treatment strategies in vivo [83]. This may be due to the current practical challenges of creating such models, and the inability of patient-derived organoids to grow in immunocompetent mice. The creation of monoclonal biallelic knockout mutant organoid lines using CRISPR is technically challenging, and variability in tumour initiation post-organoid injection into mice can pose further obstacles. For example, despite injection into syngeneic mice, Tauriello et al. [56] observed only a 30% engraftment rate of their GEMM-derived MSS organoid line, compared to the 90% tumour establishment rate in the GEMM of origin. However, higher rates of tumour initiation have been observed in other similar MSS mouse organoid studies injected into immunocompetent hosts [57]. While further optimisation is clearly required, the ability of organoid models to accurately recapitulate human CRC on multiple levels, as well as their genetic malleability establishes the importance of this technique as a key tool in CRC research. Increased use of organoids, preferably implanted at orthotopic sites, may provide relatively fast and more clinically relevant strategies for modelling ICI responses seen in CRC patients.

### Engraftment of patient-derived tissue into mice

Surgical implantation of patient-derived tumour cells, organoids or tissue into mice generates patient-derived xenograft models (PDX). This type of modelling system maintains histological characteristics as well as cellular and genetic heterogeneity of patient tumours [84–86]. As such, PDX models have been instrumental for investigations of tumour therapy responses. Indeed, Bertotti and colleagues [87] produced a xenograft screening platform of 85 human metastatic CRC samples, and found that human disease response was reliably recapitulated in the corresponding PDXs when treated with a variety of targeted therapies. Furthermore, PDX modelling has been successfully employed to investigate HER2-specific CAR-T therapy for CRC, showing promise as a preclinical modelling strategy for this type of therapy in solid tumours [88, 89]. However, PDX models have previously been limited in their use for immune checkpoint blockade due to the requirement of an immunodeficient murine host, such as NOD.Cg*-Prkdc*^*scid*^ *Il2rg*^*tm1Wjl/SzJ*^ (NSG), to avoid rejection of human tumour cells [90]. Studies have sought to overcome this obstacle through the use of immunodeficient mice with a humanised immune system following engraftment of human CD34^+^ haematopoietic stem cells (HSCs) [57, 91] or peripheral blood mononuclear cells (PBMCs). The use of PDXs in humanised mice for studying immunotherapy has been summarised previously [90], however its application to CRC immunotherapy research is in its early stages. Recently, Capasso et al. [92] developed a humanised PDX model by transplanting cord blood-derived CD34+ cells into BALB/c-Rag2^null^ Il2rγ^null^SIRPα^NOD^ mice, in which CRC PDXs were then established. When treated with anti-PD1 therapy, the growth of PDXs derived from an MSI-H CRC patient was inhibited when compared to PDXs from an MSS CRC patient, indicating a differential immune system response that mirrors what is observed in the clinic. It is important to note that while this technique partially restores the immune system, using autologous blood cells or a matched human leucocyte antigen (HLA) haplotype would provide a more accurate recapitulation of the clinical scenario. However, this would add further complexity to an already technically challenging procedure, and would potentially be unfeasible for many researchers. Practical challenges such as the expensive and time-consuming nature of these models hinder their application to real-time personalised medicine, particularly for patients with aggressive and/or advanced tumours. Further, the requirement for humanised mice in immunotherapy research (particularly access to cord blood or human foetal tissue) adds an extra layer of difficulty and cost. Most limiting is the development of graft–versus–host disease in humanised models, which can shorten the effective window to investigate therapeutic responses to 4–5 weeks. However, research in this field is rapidly evolving [93], and continued developments in medical technology will likely contribute to facilitating the establishment of humanised mouse models as powerful immunotherapeutic modelling tools.

## The role of the microbiome in CRC modelling and response to therapy

Emerging evidence has implicated the microbiome as a key player in CRC disease progression, with modulation of the TIME underlying this association, at least in part [94–96]. Studies specifically investigating the impact of the gut microbiota on immunotherapy in CRC patients are currently sparse, potentially due to the paucity of CRC patients that qualify for this type of treatment, and the small number of responders [97]. However, the effect of the microbiome specifically on the immunotherapy response has been more widely explored in cancers such as melanoma, non-small cell lung cancer and renal cell carcinoma for which ICI therapy has revolutionised treatment. Multiple studies have elucidated the regulatory roles of the gut microbiome in response to anti-PD1 immunotherapy in metastatic melanoma patients [98, 99]. Gopalakrishnan et al. [98] analysed human patient faecal microbiomes to reveal a higher gut microbiome alpha diversity and enrichment of anabolic pathways in responders to anti-PD1 immunotherapy. On further investigation using faecal microbiome transplantation mouse models, an enhanced systemic and anti-tumour immunity was also observed in mice with faecal transplants from responders. A similar study of the microbiome of hepatocellular carcinoma patients receiving anti-PD1 therapy [100] also found that responders had higher taxa richness and, within enriched species, potentially beneficial pathways such as carbohydrate metabolism and methanogenesis were upregulated. However, ‘humanising’ the mouse microbiome using FMT has elicited varied results [101, 102] due to differences in a range of variables such as processing protocol and sample type, and thus is still under continued investigation.

While CRC immunotherapy patient studies are yet to be published, the importance of elucidating the roles of the gut microbiome on the CRC TIME has been recognised by the field, particularly due to the physiological proximity of the disease to the immense array of microbes present in the gut [103]. A host of microbes, including bacteria, viruses and fungi have been shown to be enriched in CRC, which have been summarised in multiple reviews [104–107]. Some of the most studied CRC-associated microbes include genotoxic *Fusobacterium nucleatum, pks*^*+*^ *Escherichia coli* and enterotoxin-producing *Bacteroides fragilis*, with mounting evidence elucidating tumour-promoting roles for these bacteria. Incorporating an accurate representation of the tumour microbiome is therefore important in preclinical models, but also innately challenging due to the array of factors (diet, drugs, antibiotics, disease etc.) that can alter its composition [104, 108]. Furthermore, the murine gut microbiota is not necessarily conserved between strains, generations, or facilities, leading to challenges with consistency and reproducibility between experiments. There is also evidence that the microbiome of laboratory mice resembles that of immature humans, lacking effector-differentiated and mucosally distributed memory T cells [109]. To address this issue, studies have shown that cohousing laboratory mice with pet shop animals alters the microbiome, increases immune responsiveness, and elevates basal cytokine and chemokine levels [109, 110]. Thus, cohousing or pup cross-fostering arrangements, or faecal microbiota transplantation of 'wild' microbiomes, may provide a more accurate representation of mature host microbiome and ICI responsiveness in CRC models.

## Optimising preclinical CRC mouse models for investigating immunotherapies

Currently, immune-competent model systems to investigate immunotherapy options and combinations with other treatments remain limited, and translation of research findings into effective novel therapies continues to be a momentous challenge. Given the recent addition of immunotherapies to the repertoire of therapeutic options in CRC, the development of effective preclinical models is currently an unmet need. Such models need to accurately recapitulate human-like responses and elucidate anti-tumour mechanisms if we are to see improvements in the clinic based on preclinical testing. While in vitro techniques are emerging, especially the use of organoids, they are currently still limited in their ability to incorporate and maintain appropriate immune cell diversity found in vivo [111]. Furthermore, recent years have seen advancements in high-throughput single-cell omics technologies that have allowed a magnifying glass to be held up to the CRC TIME, enabling the characterisation of previously undefined cell subtypes, signalling pathways and ligand–receptor networks [112–114]. The establishment and growth of CRC is along a multi-directional axis influenced by the gene expression of the malignant cells, which shape the non-malignant cellular landscape of the tumour, and vice versa. There is an urgent need therefore to incorporate these complexities into our preclinical models, but generally speaking, with added complexity often comes lack of practicality. Many of the models we rely on today, such as cell line-derived subcutaneous models, can be critiqued for being overly reductionist, but more advanced models currently are too laborious to be considered for high-throughput screening of novel immunotherapies (Table 1).

**Table 1:** Comparison of common colorectal cancer models used for colorectal immuno-oncology research.

	Advantages	Limitations	hCRC phenotypic similarity
Chemical models	• Accurately recapitulates CAC • Straightforward and cost-effective • Can be combined with other models to decrease latency periods • Responsive to microbiome alterations	• Only represents 1–2% of CRC • Generally unresponsive to ICIs • Variability in tumour penetrance, latency and tumour antigens	CAC
Genetic models	• Develop de novo tumours gradually over time, in the presence of an intact immune system • Can investigate alteration to specific genes of interest • Can generate immune cold CRC	• Time-consuming and costly • Reduced passenger mutations • Prolonged latency periods and tumour establishment can be variable and difficult to monitor	*Apc*^*min*^: FAP *VCMsh2*^*LoxP*^: LS, MSI Next gen GEMMs: dependent on target mutations
Syngeneic cell lines	• Rapid, reproducible growth • Cost-effective and relatively straightforward • Can be injected at different anatomic sites • Can be responsive to ICI therapies	• Homogeneous tumours • Lack many common genetic and microenvironmental features of human CRC	MC38: MSI CT26: both MSI and MSS characteristics
Organoids	• Can be rapidly generated to harbour specific DNA mutations • Has the ability to be utilised both in vivo and in vitro • Can be implanted at different anatomic sites	• Creation of organoid lines is technically challenging • Some variability in tumour engraftment rates • Tumour cells do not arise de novo	Dependant on target mutations
PDXs/humanised PDXs	• Maintains histological characteristics and cellular and genetic heterogeneity of patient tumours • Allow a more patient-specific approach to therapeutic testing	• Requires an immunodeficient host mouse strain • Humanisation requires reconstitution with autologous immune cells • Expensive, complex and labourious	Dependant on subtype of xenograft

*hCRC* human colorectal cancer, *CAC* colitis-associated colorectal cancer, *ICI* immune checkpoint inhibitor, *CRC* colorectal cancer, *LS* Lynch syndrome.

**Fig. 3:**
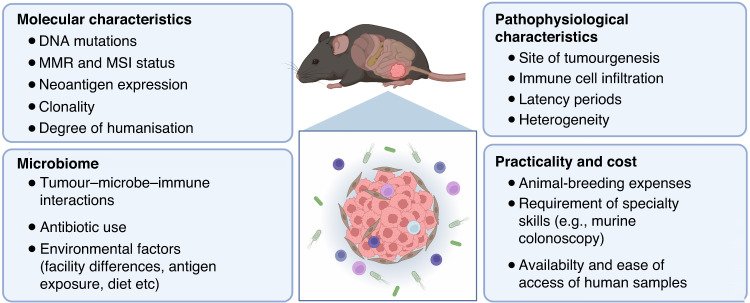
The future for immuno-oncology research using CRC mouse models. Important considerations for advancing mouse models and translational research for colorectal immuno-oncology.

Although models such as GEMMs, PDXs and organoid transplant models progress towards representing a more accurate tumour microenvironment in mice, to date there isn’t a singular 'gold standard' mouse model of CRC for immunotherapy studies. Rather, we may improve predictive power by assessing and combining the strengths of different models with important considerations, as summarised in Fig. 3, dependent on the strategy being tested [115–117]. As the majority of CRC cases are depleted of cytotoxic immune cells, this is a key feature to replicate in preclinical models to test immunotherapy strategies. In this case, a move away from immunogenic, subcutaneous cell line models, towards GEMMs and orthotopic organoid implant models, is likely to provide more accurate predictions of response. While humanised PDX models show promise, current practical challenges and high cost render them unattainable for many researchers. To continue advancing CRC preclinical modelling, a close collaboration between medical oncologists, surgeons, and cancer scientists is paramount. Cross-disciplinary studies that perform comparisons between baseline treatment-naïve tumour samples and matched post-treatment samples in responders and non-responders allow the identification of important cellular characteristics in the clinic, which can then be translated back to preclinical models for novel drug development. Such studies have begun to emerge: Zhang et al. [118] utilised scRNA-seq to investigate common myeloid subsets across both human and murine CRC, with the intent of further dissecting mechanisms that underpin immunotherapy outcomes. As these studies continue to be performed on a variety of CRC patients with distinct subtypes, the cellular landscapes and influential signalling pathways dictating ICI response will become progressively better characterised. In combination with advancing DNA manipulation technologies and therapeutic delivery strategies, there is a promising future for mouse modelling of immunotherapy for CRC.
